# Virtual primary care in high-income countries during the COVID-19 pandemic: Policy responses and lessons for the future

**DOI:** 10.1080/13814788.2021.1965120

**Published:** 2021-08-25

**Authors:** Ana Luisa Neves, Edmond Li, Pramendra Prasad Gupta, Gianluca Fontana, Ara Darzi

**Affiliations:** a Imperial NIHR Patient Safety Translational Research Centre, Institute of Global Health Innovation, Imperial College London, London, UK; b Department of General Practice and Emergency Medicine, B.P. Koirala Institute of Health Sciences, Dharan, Nepal; c Centre for Health Policy, Institute of Global Health Innovation, Imperial College London

**Keywords:** Primary care, health policy, COVID-19, telemedicine, health informatics, clinical informatics

## Abstract

**Background:**

Telemedicine, once defined merely as the treatment of certain conditions remotely, has now often been supplanted in use by broader terms such as ‘virtual care’, in recognition of its increasing capability to deliver a diverse range of healthcare services from afar. With the unexpected onset of COVID-19, virtual care (e.g. telephone, video, online) has become essential to facilitating the continuation of primary care globally. Over several short weeks, existing healthcare policies have adapted quickly and empowered clinicians to use digital means to fulfil a wide range of clinical responsibilities, which until then have required face-to-face consultations.

**Objectives:**

This paper aims to explore the virtual care policies and guidance material published during the initial months of the pandemic and examine their potential limitations and impact on transforming the delivery of primary care in high-income countries.

**Methods:**

A rapid review of publicly available national policies guiding the use of virtual care in General Practice was conducted. Documents were included if issued in the first six months of the pandemic (March to August of 2020) and focussed primarily on high-income countries. Documents must have been issued by a national health authority, accreditation body, or professional organisation, and directly refer to the delivery of primary care.

**Results:**

We extracted six areas of relevance: primary care transformation during COVID-19, the continued delivery of preventative care, the delivery of acute care, remote triaging, funding & reimbursement, and security standards.

**Conclusion:**

Virtual care use in primary care saw a transformative change during the pandemic. However, despite the advances in the various governmental guidance offered, much work remains in addressing the shortcomings exposed during COVID-19 and strengthening viable policies to better incorporate novel technologies into the modern primary care clinical environment.

KEY MESSAGES

- COVID-19 has tested the capacity of pre-existing remote models of care, both as a complementary and alternative means of delivering community-based healthcare.
- Virtual care policies implemented in high-income countries successfully facilitated the delivery of remote preventive and acute care, as well as safe triage processes.
- This emergency response had also highlighted concerns regarding current telemedicine security standards and the need for new modes of funding and reimbursement.

## Introduction

Digital technology has transformed many aspects of modern life; healthcare is no exception. Over the last decade, primary care systems have slowly started to adopt virtual modes of delivery, in which digital tools (i.e. telephone, online video) serve as a first point of contact for patients, directing them to the appropriate digital or face-to-face services based on their needs [1,2]. This approach can provide access to a range of primary care services, such as booking and cancelling appointments, having remote consultations, receiving referrals, and obtaining prescriptions [1,3]. As part of a streamlined, integrated experience, ‘virtual approaches’ have the potential of improving efficiency, patient safety, and access to care [4]. Alongside this gradual transformation, saw a shift in the accompanying terminology with which to describe the underlying technology. The term ‘telemedicine’ which once was defined strictly as the treatment of certain conditions remotely, has now given way somewhat to broader terms such as ‘virtual care’ – a testament to the rapidly expanding functionality and application of these digital tools to facilitate the delivery of more holistic care remotely [5]. For this reason, in this rapid review the latter term was chosen.

As a response over the last several years, health policies have been preparing to incorporate virtual care as an essential part of health care delivery. For example, NHS England declared in 2019 that all patients should have the right to video consultations by 2021 and that all primary care practices should ensure at least 25% of their appointments are available for online booking [6]. However, despite similar statements observed worldwide, adoption remained slow, in part due to a general hesitance with novel technologies, privacy concerns, limited stakeholder enthusiasm, and inadequate investment [7,8].

By examining publicly available national guidance documents available to GPs, this background paper aims to examine the differing policy-based approaches taken to meet this challenge, potential technical shortcomings, and ultimately, its effects on revolutionising the delivery of primary care.

## Methods

In this background paper, we adopted the principles of a rapid review to identify key areas of relevance on this topic. Rapid reviews are a form of knowledge synthesis in which components of the systematic review process are simplified to produce information on time [9]. In light of the rapidly evolving pandemic, policymakers require evidence synthesis to produce robust guidance for primary care providers. The World Health Organisation (WHO) recommends rapid reviews to provide such evidence [9].

To identify relevant documents, we have searched the websites of relevant national departments and health authorities (ministries of health, primary care organisations and regulatory bodies). In what concerns the inclusion criteria adopted, documents were included if issued in the first six months of the pandemic (March to August of 2020) and focussed on using remote care tools in high-income countries. Documents must have been issued by a national health authority, accreditation bodies or professional organisation, and directly refer to the delivery of primary care. The documents were subsequently evaluated by two independent researchers, with the findings included this rapid review derived from them reaching a consensus upon regular discussions.

## Results

Our rapid literature review has identified 16 documents from nine countries (Australia, Bosnia, Canada, Germany, Italy, Netherlands, Spain, United Kingdom, and the United States). From those, we extracted six areas of relevance: primary care transformation during COVID-19, the continued delivery of preventative care, the delivery of acute care, remote triaging, funding & reimbursement, and roadmap for the future of remote care models.

### COVID-19 and the primary care transformation

With the COVID-19 pandemic, the digital landscape in primary care was about to experience a dramatic change. On 11 March 2020, the World Health Organisation officially declared COVID-19 a global pandemic [10]. In the United Kingdom, GPs were hastened to shift patients over to online and electronic prescription services for the dispensing of medications [11]. In Australia, a rapid transition to using mainly virtual consultations was deemed appropriate for most patients who have visited their GPs at least once in the past 12 months or those who specifically requested care to be continued remotely [12]. This new reality, with drastic limitations curtailing physical contact, has resulted in virtual solutions taking centre stage in primary care delivery [13]. In many ways, the COVID-19 outbreak has presented a unique opportunity that has both tested the capacity of pre-existing virtual models of care, and simultaneously demonstrated their growing importance as a complementary and alternative means of delivering community-based healthcare. Virtual models of care have also been increasingly relied upon to coordinate and support public health responses to the pandemic itself, all the while minimising risks of exposure for patients, healthcare providers, and the public [3,13–15].

As part of this transformation, health policies worldwide have undergone drastic changes to adapt and facilitate virtual models’ upscaling. While these changes have lowered many of the existing barriers to digital healthcare, it is important to reflect upon their impact not just as an immediate solution to a public health emergency but also as the first of many potential regulatory adjustments necessary to enabe the continuation and proliferation of high-quality, safe virtual models usage in primary care in the future.

### Delivery of routine and preventative care

Proactive health promotion and preventive care are an integral part of primary care. During a pandemic, these services still need to be delivered. The US Centres for Disease Control and Prevention (CDC) now supports the use of remote care in a range of routine and preventive care services, including management of chronic health conditions, monitoring of clinical signs (*i.e.* blood pressure, blood glucose, other remote assessments), patient coaching and support (*i.e.* weight management and nutrition counselling), and medication management [16]. In Australia, Canada, and the United Kingdom, new guidance has been provided advising GPs to shift patients whenever possible, over to online or telephone-based consultations [12,17,18]. In Germany, the recently passed Digital Healthcare Act in conjunction with the ongoing COVID-19 pandemic, is now used as a catalyst to democratise the routine use of video-based consultations previously requiring an in-person visit first with the clinician [19]. Former insurance reimbursement restrictions capping the proportion of cases seen by clinicians *via* telemedicine, as well as what types of consultations services could be provided, have also been lifted [19].

In the context of providing routine and preventative care, ‘digital-first’ approaches can be valuable tools to maintain continuity, improve timeliness, and mitigate the negative consequences of delays on provision of care [16]. Additionally, these approaches may also be beneficial in preserving the patient-physician relationship at a time when face-to-face visits are not safe, and facilitate the engagement of those who are shielding, have limited mobility or other physical limitations to access care [16].

At the same time, we must be aware of some less understood risks. When monitoring clinical signs and symptoms remotely, not all patients have access to medical-grade, home-based self-monitoring devices (*i.e.* BP monitor, blood glucose monitor, weighing scale), and may not necessarily be familiar with proper operating procedures [20]. We cannot risk leaving many of the most vulnerable patients excluded from preventive care or further alienated by the digital divide. Future policies promoting the use of virtual models for routine and preventive care must consider these disparities in the engagement with digital care, which are often driven by ethnicity, age, and socioeconomic status. Left unaddressed, these disparities can lead to the further widening of health inequities [21]. Health policies must therefore incorporate more inclusive implementation strategies by comprising the perspectives of both healthcare providers and patients alike, and strengthen telehealth training to accommodate for language and cultural barriers, varying levels of digital literacy, and disability [21].

### Delivery of acute care

Additionally, virtual approaches can also be used to provide low-risk acute care for non-COVID-19 conditions in the community, identify those persons who may need additional medical consultation or assessment, and refer them as appropriate [16]. However, such substantial change to the traditional means of conducting a clinical consultation inevitably brings with it challenges, including the impracticality of conducting certain acute consultations requiring specialised equipment or physical examination and utilising a simple, reliable means of audio-visual communication with a diverse range of patients. In these circumstances, virtual care can have a detrimental effect on diagnostic uncertainty, a particularly relevant feature in primary care due to the breadth and complexity of diagnoses possible, and its implications on patient safety [22].

To mitigate the associated risks, the US CDC developed the ‘Framework for Healthcare Systems Providing Non-COVID19 Clinical Care’, to assist healthcare providers in determining when in-person acute care is appropriate [23]. Similarly, UK, Canadian, and Australian national accreditation bodies also included comprehensive guidelines defining what tasks are appropriate to be performed *via* virtual models, what equipment is required, and most importantly, how GPs should go about conducting consultations remotely [12,20,24]. When incorporated in telemedicine policies guidance and materials, the availability of such clinical decision-support tools is key to continue providing necessary services while minimising the risk of harm to patients and providers.

### COVID-19 remote triaging

During the COVID-19 outbreak, virtual models have also been used in a triaging capacity in primary care to identify new cases in the community, which patients may require further testing and potentially helping to curtail spread in the wider population [25,26]. The national guidance documentation of Australia, Canada, and the United Kingdom, detailed how to remotely assess patients who present with symptoms suggestive of COVID-19, what public health resources are available, and the appropriate next course of action to take [12,20,24]. In Germany, this was further accompanied by the rollout of patient self-assessment tools to optimise the use of existing telemedicine capacity and enable the ability to detect potential COVID-19 cases early on [27].

In the United States, automated bots have been incorporated alongside existing telemedicine services to allow the triaging of a rapidly expanding number of patients [26]. Patients with symptoms suggestive of COVID-19 infection and deemed higher risk, were automatically referred for further triaging in hospitals. Those with lower risk presentations were scheduled for consultations *via* telemedicine remotely, thus streamlining the workload for GPs, minimising the need for patients to present to hospital, and reducing overall risks of exposure [26]. As digital triaging permeates to other clinical settings and countries in the future, further evaluation of its impact on quality of care, and particularly on patient safety and efficiency of care delivery, is needed.

However, it is important to note that there is no standardised, validated tool to perform remote assessment of patients with COVID-19, nor to stratify their risk of clinical deterioration. The NEWS2 score, an early warning score recommended by the UK’s National Institute for Health and Care Excellence (NICE) in its guidelines for managing COVID-19 patients in critical care, has not been validated in primary care, nor for triage purposes [20]. Further research should pave the way to development of more bespoke prognostics scores to be used in the remote assessment of COVID-19 patients in primary care, capitalising on the growing body of data and novel data analytics methods available [28].

### Technology suppliers and security standards

During the first wave of the COVID-19 outbreak, many national policies have loosened security standards and relaxed telemedicine restrictions, with several high-income countries permitting the use of common, off-the-shelf communications software to ensure a quick transition for both GPs and their patients. In the US, the Department of Health and Human Services (HHS) has waived penalties regarding the use of video consultation software not previously approved to meet Health Insurance Portability and Accountability Act (HIPAA) requirements, allowing widely accessible consumer-grade services such as FaceTime or Skype to be used for telemedicine purposes, even if the care service is not related to COVID-19 [29]. In Europe, the General Data Protection Regulations already include a clause exempting work in the overwhelming public interest. In the UK, NHSx encourages videoconferencing tools such as Skype, WhatsApp, Facetime, or other commercial products designed specifically for healthcare purposes [30].

Although these policies have acted as strong drivers to expanding virtual care availability during the pandemic, the relaxation of privacy standards raises concerns, including the possibility that patients’ data shared over a non-compliant platform may be inappropriately accessed, shared, or monetised. To address these risks and maintain the momentum needed to building safer solutions, national guidance from several countries such as the Netherlands, US, and the UK, do include a well-defined list of pre-approved vendors [31–33]. In the absence of a list of pre-approved software vendors, Canadian and Australian national accreditation body guidelines provided additional support for GPs specifying how to set up their workplace in a secure manner and remotely take patient informed consent to safeguard patient privacy [12,34].

### Funding and reimbursement

The first wave of COVID-19 demonstrated the need to streamline and reinforce existing funding avenues to answer the need for virtual care delivery during a pandemic. In this context, governments and health policymakers worldwide have unveiled guidance regarding changes in existing billing procedures and the availability of additional funding, with important implications on how primary care is delivered and funded. For example, in Australia, novel financial initiatives such as doubling of ‘bulk billing’ have been put in place [12]. Guidance into what types of patients, and what procedures, items, and services were eligible for the new billing arrangements, was extensively detailed in the new telemedicine guidelines [12,35]. In Canada, several provincial health authorities have introduced new telemedicine-specific billing codes in the hopes of simplifying the overall transition process [34,36].

Additionally, the COVID-19 outbreak raised awareness on the importance of strategically investing in ‘digital-first’ programmes. These programmes represent not only an emergency response to the crisis but also a potential long-term strategic vision to addressing a multitude of existing needs in primary care delivery, particularly reaching out to patients with poor access to care. In the US, the newly passed ‘Coronavirus Aid, Relief, and Economic Security (CARES) Act’ awards a total of $8.7 million a year for telehealth technologies used in rural areas and medically underserved areas [37]. Recent changes to Medicare as well as initiatives by some private insurers have permitted consultations completed *via* telemedicine to be paid at the same rate as those conducted in-person [38]. However, it remains to be seen as to whether these changes will persist after the eventual passing of COVID-19 and translate into a more fundamental transformation of how virtual care is funded [19].

## Discussion

### Implications for the long-term future

The COVID-19 pandemic has presented a unique opportunity to challenge the long-established relationship between virtual approaches and their role in community-based care. The emergent circumstances have undoubtedly allowed many of the barriers to change to be overcome. However, this abrupt scaling up of telemedicine has also exposed many weaknesses in existing virtual solutions. It has highlighted the need for health policies to guide the allocation of limited resources better, greater investments to modernise infrastructure, expansion of technical training, prioritisation for systems interoperability [39], and more support for patients with lower health and digital literacy levels [40].

While the pandemic represents an opportunity to rethink how virtual care can be better integrated into primary care, it is imperative to address these challenges through clear, deliberate policies to ensure that this devastating pandemic leaves a positive legacy. Policymakers and researchers need to establish strategic partnerships and undertake a rigorous evaluation of what worked, for which patients, and in what clinical context, to draw lessons for the long-term and define evidence-based policies.

Finally, primary healthcare policies need to be judiciously designed rather than wishfully conceived – an often-underestimated aspect in current processes [41]. Policies must be able to bridge the gap between theory and practice, both by proposing realistic models and providing the necessary support to translate primary care objectives into reality [41,42]. The inherent complexity of modern primary health systems and their governance makes it unlikely that policies can be designed *a priori* perfectly, without necessitating continuous, iterative improvement. Therefore, a greater emphasis on sound policy design, holistically incorporating healthcare staff and patients’ feedback on design and maintenance, is key to ensuring the planned actions allow for innovative yet pragmatic means of achieving policy goals.

### Limitations

It must be acknowledged that this background paper consisted in a rapid review of the literature available from the first months of the pandemic to provide a first overview of the subject. Given the rapidly changing nature of the pandemic, many of the guidance documentation examined were temporary measures hastily introduced and likely subject to further refinement and subsequent changes. It is likely that new guidance has been published since and would require a follow-up re-examination to evaluate more comprehensively the breath of new information available on this evolving subject.

It is also important to note that rapid reviews are a preferred option when health decision-makers need timely access to information for background purposes, as per the aim of this background paper. However, they may produce less reliable evidence and may lead to suboptimal decision-making. As future steps for researchers, we suggest further research should evaluate the evidence available on this subject systematically, adhering to the principles and high-level of rigour of a systematic review, including systematic searches and screening, data abstraction, and risk of bias appraisal conducted by two individuals, independently [43].

## Conclusion

The COVID-19 outbreak has been a litmus test for the robustness of virtual care models’ implementation in primary care. It has both revealed its many shortcomings yet simultaneously allowed for novel ideas tackling some of the deep-seated problems to be explored. It is now time to incorporate the practical lessons learned and reshape current policies, rules, and regulations to support safer, more efficient, and more equitable use of virtual care solutions.

## References

[1] NHS Digital. NHS England » Digital First Primary Care. 2019; [cited 2020 Nov 24]. Available from: https://www.england.nhs.uk/gp/digital-first-primary-care/.

[2] Bokolo AJ. Exploring the adoption of telemedicine and virtual software for care of outpatients during and after COVID-19 pandemic. Ir J Med Sci. 2021;190(1):1–10. 10.1007/s11845-020-02299-z PMC7340859 32642981

[3] Monaghesh E, Hajizadeh A. The role of telehealth during COVID-19 outbreak: a systematic review based on current evidence. BMC Public Health. 2020;20(1):1193–1199. 32738884 10.1186/s12889-020-09301-4 PMC7395209

[4] O'Connor M, Asdornwised U, Dempsey ML, et al. Using telehealth to reduce all-cause 30-day hospital readmissions among heart failure patients receiving skilled home health services. Appl Clin Inform. 2016;7(2):238–247. 27437037 10.4338/ACI-2015-11-SOA-0157 PMC4941836

[5] Health T. Telemedicine vs. Virtual Care: defining the difference. 2020; [cited 2021 July 5] Available from: https://intouchhealth.com/finding-the-right-term-for-modern-digital-healthcare/?gdprorigin=true.

[6] National Health Service (NHS). NHS Long term plan. NHS Engl. 2019; [cited 2020 Nov 24] Available from: https://www.longtermplan.nhs.uk/.

[7] Harvey JB, Valenta S, Simpson K, et al. Utilization of outpatient telehealth services in parity and nonparity states 2010–2015. Telemed J E Health. 2019;25(2):132–136. 29847224 10.1089/tmj.2017.0265

[8] Krelle H, Dodson JA, Horwitz L. Virtual primary care – is its expansion due to COVID-19 all upside? JAMA Heal Forum. 2020;1(7):e200900. 10.1001/jamahealthforum.2020.0900 36218702

[9] Tricco AC, Langlois EV, Straus SE. Rapid reviews to strengthen health policy and systems: a Practical Guide (World Health Organization-WHO 2017). *WHO* Published Online First: 2018; [cited 2021 May 24]. Available from: http://www.who.int/alliance-hpsr/resources/publications/rapid-review-guide/en/.

[10] World Health Organization (WHO). WHO Director-General’s opening remarks at the media briefing on COVID-19 – 11 March 2020. WHO Dir Gen speeches 2020. 4; [cited 2020 Nov 26]. Available from: https://www.who.int/director-general/speeches/detail/who-director-general-s-opening-remarks-at-the-media-briefing-on-covid-19–-11-march-2020.

[11] NHS England. Electronic repeat dispensing: Letter from Dr Nikita Kanani, Dr Keith Ridge and Ed Waller: 4 June 2020. 2020; [cited 2020 Dec 22]. Available from: https://www.england.nhs.uk/coronavirus/wp-content/uploads/sites/52/2020/03/C0546-electronic-repeat-dispensing-letter-4-june-2020.pdf.

[12] The Royal Australian College of General Practitioners. Guide to providing telephone and video consultations in general practice. 2020; [cited 2020 Nov 24]. Available from: www.racgp.org.

[13] Anthony Jnr B. Use of telemedicine and virtual care for remote treatment in response to COVID-19 pandemic. J Med Syst. 2020;2020:44. 10.1007/s10916-020-01596-5 PMC7294764 32542571

[14] Car J, Koh GCH, Foong PS, et al. Video consultations in primary and specialist care during the covid-19 pandemic and beyond. Br Med J. 2020;2020:371. 10.1136/bmj.m3945 33082127

[15] Anthony Jnr B. Implications of telehealth and digital care solutions during COVID-19 pandemic: a qualitative literature review. Inform Health Soc Care. 2021;46(1):68–83. 33251894 10.1080/17538157.2020.1839467

[16] Centers for Disease Control and Prevention (CDC). Using telehealth to expand access to essential health services during the COVID-19 pandemic. Centers Dis Control Prev. 2020;2019:2–5.

[17] Health M. A toolkit for building and growing a sustainable telehealth program in your practice. Published Online First: 2020; [cited 2020 Nov 24]. Available from: https://www.manatt.com/Health.

[18] NHS England. Millions of patients benefiting from remote consultations as family doctors respond to COVID-19. News. 2020;1:19–21.https://www.england.nhs.uk/2020/05/millions-of-patients-benefiting-from-remote-consultations-as-family-doctors-respond-to-covid-19/

[19] Gerke S, Stern AD, Minssen T. Germany’s digital health reforms in the COVID-19 era: lessons and opportunities for other countries. NPJ Digit Med. 2020;3:1–6. 32685700 10.1038/s41746-020-0306-7 PMC7351985

[20] Royal College of General Practitioners. Principles for supporting high quality consultations by video in general practice during COVID-19. Published Online First: 2020; [cited 2020 Nov 24]. Available from: https://www.england.nhs.uk/coronavirus/wp-content/uploads/sites/52/2020/03/C0479-principles-of-safe-video-consulting-in-general-practice-updated-29-may.pdf.

[21] Gray DM, Joseph JJ, Olayiwola JN. Strategies for digital care of vulnerable patients in a COVID-19 world—keeping in touch. JAMA Heal Forum. 2020;1(6):e200734. 10.1001/jamahealthforum.2020.0734 36218529

[22] Dicker A. Should doctors observe a moral duty to care for themselves? In: Primary care ethics. Boca Raton: CRC Press; 2018. p. 137–147.

[23] Centers for Disease Control and Prevention (CDC). Non-COVID-19 Care Framework | CDC. 2020; [cited 2020 Nov 24]. Available from: https://www.cdc.gov/coronavirus/2019-ncov/hcp/framework-non-COVID-care.html.

[24] The College of Family Physicians of Canada, Royal College of Physicians and Surgeons of Canada, Canadian Medical Association. Virtual Care Playbook. 2020.

[25] Hur J, Chang MC. Usefulness of an online preliminary questionnaire under the COVID-19 pandemic. J Med Syst. 2020;44(7):116. 32430783 10.1007/s10916-020-01586-7 PMC7235540

[26] Hollander JE, Carr BG. Virtually perfect? Telemedicine for covid-19. N Engl J Med. 2020;382(18):1679–1681. 32160451 10.1056/NEJMp2003539

[27] Peine A, Paffenholz P, Martin L, et al. Telemedicine in Germany during the COVID-19 pandemic: multi-professional national survey. J Med Internet Res. 2020;22(8):e19745. 32568724 10.2196/19745 PMC7409912

[28] Greenhalgh T, Thompson P, Weiringa S, et al. What items should be included in an early warning score for remote assessment of suspected COVID-19? qualitative and delphi study. BMJ Open. 2020;10(11):e042626. 10.1136/bmjopen-2020-042626 PMC7662139 33184088

[29] U.S. Department of Health and Human Services. Notification of Enforcement Discretion for Telehealth Remote Communications During the COVID-19 Nationwide Public Health Emergency. HHS.gov Published Online First: 2020; [cited 2020 Nov 24]. Available from: https://www.hhs.gov/about/news/2020/03/17/ocr-announces-notification-of-enforcement-discretion-for-telehealth-remote-communications-during-the-covid-19.html.

[30] NHSX. COVID-19 IG advice; [cited 2020. Nov 24]. Available from: https://www.nhsx.nhs.uk/information-governance/guidance/covid-19-ig-advice/.

[31] University of Arizona. Service Provider Directory. 1876. 11–2; [cited 2020 Nov 24]. Available from: https://telemedicine.arizona.edu/servicedirectory.

[32] National Organization of State Offices of Rural Health. Telehealth Technologies and Preparing to Select a Vendor; [cited 2020. Nov 24]. Available from: https://nosorh.org/wp-content/uploads/2016/11/NOSORH-Telehealth-Vendor-Fact-Sheet-FINAL.pdf.

[33] NHS England and NHS Improvement. Procurement of pre-approved suppliers of online and video consultation systems for GP practices to support COVID-19. 2020; [cited 2020 Nov 24]. Available from: https://www.england.nhs.uk/digitaltechnology/digital-primary-care/commercial-procurement-hub/dynamic-purchasing-system/.

[34] Royal College of Physicians and Surgeons of Canada. Telemedicine and virtual care guidelines for health professionals. R. Coll. Physicians Surg. Canada. 2020; [cited 2020 Nov 24]. Available from: https://www.royalcollege.ca/rcsite/documents/about/covid-19-resources-telemedicine-virtual-care-e.

[35] Australian Government Department of Health. Coronavirus (COVID-19) – Telehealth items guide; [cited 2020. Nov 24]. Available from:http://www.mbsonline.gov.au/internet/mbsonline/publishing.nsf/.

[36] Canadian Institute for Health Information (CIHI). Physician billing codes in response to COVID-19 | CIHI. 2020;. [cited 2020 Nov 24]. Available from: https://www.cihi.ca/en/physician-billing-codes-in-response-to-covid-19.

[37] Courtney J. H.R.748 – 116th Congress (2019-2020): CARES Act. 2020. [cited 2020 Nov 24]. Available from:https://www.congress.gov/bill/116th-congress/house-bill/748.

[38] CMS. SPECIAL EDITION Special Edition of MLN Connects. 2020; [cited 2020 Nov 24]. Available from: https://www.cms.gov/files/document/mln-connects-special-edition-3-31-2020.pdf.

[39] Neves A, Lygidakis H, Fontana G. The technology legacy of COVID-19 in primary care. bjgplife.com. Published Online First: 2020; [cited 2020 Nov 24]. Available from: https://bjgplife.com/2020/04/15/the-technology-legacy-of-covid-19-in-primary-care/.

[40] Donaghy E, Atherton H, Hammersley V, et al. Acceptability, benefits, and challenges of video consulting: a qualitative study in primary care. Br J Gen Pract. 2019;69(686):e586–94. 31160368 10.3399/bjgp19X704141 PMC6617540

[41] Hallsworth M, Parker S, Rutter J. Policy-making in the real world. Polit Insight. 2011;2(1):10–12.

[42] Sinsky CA. Implementing telemedicine in primary care: Learning lessons from electronic Health Records. Mayo Clin Proc. 2020;95(9):1835–1837. 32861325 10.1016/j.mayocp.2020.07.017 PMC7449897

[43] Higgins JP, Green S. Cochrane handbook for systematic reviews of interventions | cochrane training. Handbook. 2011;2:649. https://training.cochrane.org/handbook/current

